# Supplementary material for: Ecology of the digital world of Wikipedia
Source: Sci Rep. 2021 Sep 15;11:18371. doi: 10.1038/s41598-021-97755-w (PMC8443573; doi:10.1038/s41598-021-97755-w)
Supplement: Supplementary file 1 — Supplementary Information. [file 41598_2021_97755_MOESM1_ESM.pdf]

Supplementary Information for “Ecology of the digital world of Wikipedia”

by Fumiko Ogushi, János Kertész, Kimmo Kaski, and Takashi Shimada*

Network characteristics

Degree and Strength

We denote the degree of editor ε and article α as

$$k^E_\varepsilon = \sum_\alpha b_{\alpha\varepsilon}, \quad k^A_\alpha = \sum_\varepsilon b_{\alpha\varepsilon}, \tag{1}$$

and the strength of those as

$$s^E_\varepsilon = \sum_\alpha w_{\alpha\varepsilon}, \quad s^A_\alpha = \sum_\varepsilon w_{\alpha\varepsilon}. \tag{2}$$

Measuring Nestedness

In order to characterize the nestedness of our Wikipedia edit-network, we need a measure that is independent of degree distribution. For this reason, we adopt a nestedness measure, which was proposed for ecological networks[1]. For our network $a_{\varepsilon\alpha} = B_{\varepsilon\alpha}$ for the binary case and $a_{\varepsilon\alpha} = W_{\varepsilon\alpha}$ for the weighted case, the (local) nestedness of the edits made by editor i and that made by editor j is defined as follows

$$\eta^E_{ij} = \frac{\sum_{\alpha=1}^{N_A} a_{i\alpha}a_{j\alpha}}{k^E_i k^E_j}, \tag{3}$$

which basically counts the overlap between these two edit patterns. In the same manner, the nestedness between the edit patterns on article i and j reads

$$\eta^A_{ij} = \frac{\sum_{\varepsilon=1}^{N_E} a_{\varepsilon i}a_{\varepsilon j}}{k^A_i k^A_j}. \tag{4}$$

Note that the degree of editors and articles (k^E_i, k^A_j) in the equations above should be substituted by the corresponding strength (s^E_i, s^A_j) for the weighted network W. Then the local nestedness of an editor and an article are defined as the averages for those nodes as

$$\eta^E_i = \frac{\sum_{j\neq i}^{N_E} \eta^E_{ij}}{N_E - 1}, \qquad \eta^A_i = \frac{\sum_{j\neq i}^{N_A} \eta^A_{ij}}{N_A - 1}. \tag{5}$$

Then the global nestedness of the network is characterized by the averages of the editors' nestedness and the articles' nestedness:

$$\eta^E = \frac{\sum_{i,j\neq i}^{N_E} \eta^E_{ij}}{(N_E - 1)N_E}, \qquad \eta^A = \frac{\sum_{i,j\neq i}^{N_A} \eta^A_{ij}}{(N_A - 1)N_A}. \tag{6}$$

A merit on taking this measure is that the baseline nestedness, which is defined as the nestedness of the network with no correlation among the links, keeping the degree distribution (configuration model)

$$a_{ij} = \frac{k^E_i k^A_j}{\langle k_E \rangle N_E} = \frac{k^E_i k^A_j}{\langle k_A \rangle N_A}, \tag{7}$$

*shimada@sys.t.u-tokyo.ac.jp

can be simply (analitycally) calculated as

$$\bar{\eta}^{E_C} \equiv \langle \eta_i^{E_C} \rangle = \left(\frac{1}{N_A} \right) \frac{\langle k_A^2 \rangle}{\langle k_A \rangle^2}, \qquad \eta^{\bar{A}_C} \equiv \langle \eta_j^{A_C} \rangle \left(\frac{1}{N_E} \right) \frac{\langle k_E^2 \rangle}{\langle k_E \rangle^2}. \tag{8}$$

The global nestednes relative to this baseline,

$$\tilde{\eta}^E = \frac{\eta^E}{\bar{\eta}^{E_C}}, \qquad \tilde{\eta}^A = \frac{\eta^A}{\bar{\eta}^{A_C}}, \tag{9}$$

are found to be larger than 1 in the Wikipedia network (Table 1 in the main text). This tells that it is positively nested (i.e. the edit pattern is positively correlated).

The effect of trimming the low-degree articles

The number of editors working on the article (i.e. article degree) influences the goodness of the article. Although the contribution of the editors on the articles can be largely different, the low-degree articles tend to contain poor information. We thus set the trimming threshold for the article degree. The network with threshold x is the network after trimming the articles with degree $k_a \leq x$. The network with threshold 0 is the original network.

To assess the effect of the trimming threshold on our self-consistent analysis, we calculate the correlation of the complexity measure between the networks with different threshold values. The Pearson's correlation coefficient p_{xy} of the complexity measure between two networks with threshold value x and y is defined as,

$$p_{xy} = \frac{\sum_{\alpha}^{all}(C_{\alpha}^{x} - \langle C^{x}\rangle)(C_{\alpha}^{y} - \langle C^{y}\rangle)}{\sqrt{\sum_{\alpha}^{all}(C_{\alpha}^{x} - \langle C^{x}\rangle)^2}\sqrt{\sum_{\alpha}^{all}(C_{\alpha}^{y} - \langle C^{y}\rangle)^2}}, \tag{10}$$

where C_{α}^{x} and C_{α}^{y} denote the complexity of the article α of the network with threshold x and y, respectively. The average complexity of the network is, by definition, $\langle C^{x}\rangle = \langle C^{y}\rangle = 1$. The Spearman's rank correlation coefficient ρ_{xy} is given by the Pearson's correlation coefficient calculated on the ranks of the data instead of the original values,

$$\rho_{x,y} = \frac{\sum_{\alpha}^{all}(R_{C_{\alpha}^{x}} - \langle R_{C^{x}}\rangle)(R_{C_{\alpha}^{y}} - \langle R_{C^{y}}\rangle)}{\sqrt{\sum_{\alpha}^{all}(R_{C_{\alpha}^{x}} - \langle R_{C^{x}}\rangle)^2}\sqrt{\sum_{\alpha}^{all}(R_{C_{\alpha}^{y}} - \langle R_{C^{y}}\rangle)^2}}, \tag{11}$$

where $R_{C_{\alpha}^{x}}$ and $R_{C_{\alpha}^{y}}$ denote the complexity rank of the article α of the network with threshold x and y, respectively. $\langle R_{C^{x}}\rangle$ and $R_{C^{y}}$ denote the average rank. As shown in Fig. 6, there are three different fixed points, the networks with threshold $0 \sim 2$, the networks with threshold $3 \sim 6$, and the networks with threshold ≥ 7.

Top-N hit rate for finding the "fetured" articles depends on the threshold value. The finding accuracy does not monotonically increase with increasing the threshold value as shown in the right panel in Fig. 6. The original network skims "featured" articles faster than the network with threshold 1. As increasing the threshold value from 1 to 19, the accuracy increases for the networks with small threshold but decreases for the networks with large threshold. The network with threshold 9 and 10 work best. We use the optimal threshold 9 for our self-consistent analysis.

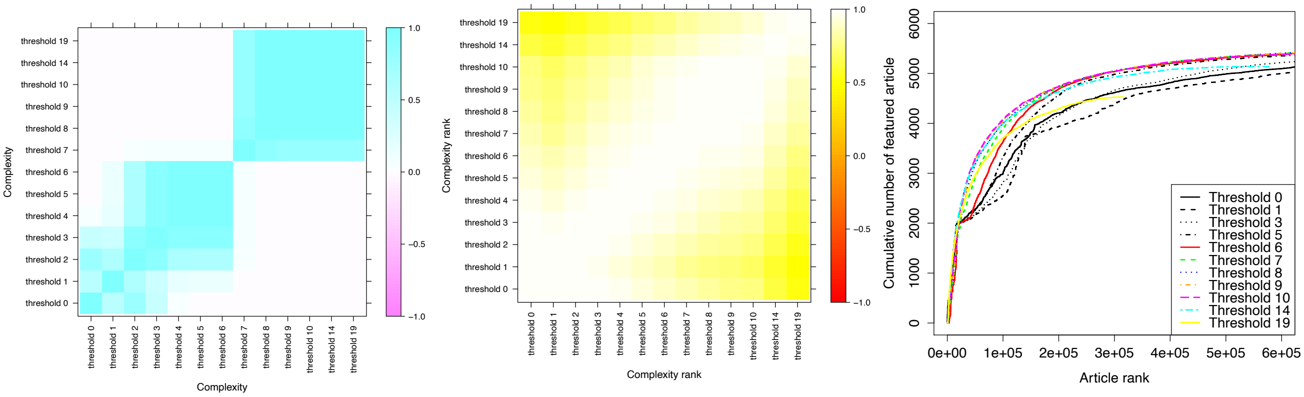

Figure 6. The effect of the trimming threshold. (Left) Person's correlation coefficient of the complexity measure. (Middle) Spearman's rank correlation of the complexity rank. (Right) Cumulative number of "featured" articles contained in the top-ranked articles in complexity measure for different threshold values. To find the "featured" articles, the networks after trimming the articles with degree $k_a \leq 9$ (orange) and $k_a \leq 10$ (magenta) work best.

Relation to the metric of country fitness and product complexity

Our formulation is inspired by the self-consistent measure proposed for countries' economic fitness F and product's complexity Q[2,3] for the world trade network, calculated as

$$\tilde{F}_i^{(n+1)} = \sum_{\alpha} b_{i\alpha} Q_{\alpha}^{(n)}, \quad \tilde{Q}_j^{(n+1)} = \left(\sum_{\varepsilon} \frac{b_{\varepsilon j}}{F_{\varepsilon}^{(n)}} \right)^{-1}. \tag{12}$$

The reason why we can not simply adopt the original definition of quality for the article complexity is the following. At a first glance, one could be inclined to regard the editors as the countries and the articles as the products, naturally from its causality relation: editors write the articles and not vice versa. However, on the Wikipedia network, there is no *capability* which limits the touch of editors to an articles (e.g. everyone could make an edit on an article of quantum physics and mid-century history of a certain local village of Japan). Therefore the selectiveness of the editors editing an article is not a good measure of its complexity or goodness, meaning that this way of straight forward application is not appropriate for Wikipedia network.

On the contrary, the selectiveness of the opponent articles in the Wikipedia bipartite network gives good information about the editors. Because we here take the top-editors, all of them are editing on thousands of articles. Some of the edits are contents edit and some can be more maintenance type edits such as small or systematic correction. Although it is hard to distinguish these maintenance like activity from the edit size or other information, we can expect that an editor editing so many articles (some indeed edit millions), especially including articles with low "goodness" (complexity), has a lower probability to make a contents edit contributing the "goodness" of articles. The selective edit records mainly on "good" articles, on the other hand, can be regarded as a good indication of the higher contents writing contribution to the article by his each edit. This type of character of editors, not the "fitness" of it, is measured by the *scatterdness* in our framework.

Note that for binary network $b_{\varepsilon\alpha}$, our definition is equivalent to taking the inverse of the products' complexity index as new complexity index for articles in the original definition for economy relation (Eqs. (12)), i.e. $C_j = Q_j^{-1}$, and therefore essentially the same as taking the inverse rank for the complexity in the original definition, except for the normalization condition. In the present work, we take weighted network $w_{\varepsilon\alpha}$ mainly because of its better performance as shown in Fig. 7 and hence this direct relation is lost. However, the mapping relation for the binary network still gives a good guide for considering the convergence of our non-linear recursion process to a non-trivial fixed point with smooth distribution of the resulting values, thanks to the intensive work on the convergence condition for the fitness-complexity measure[4].

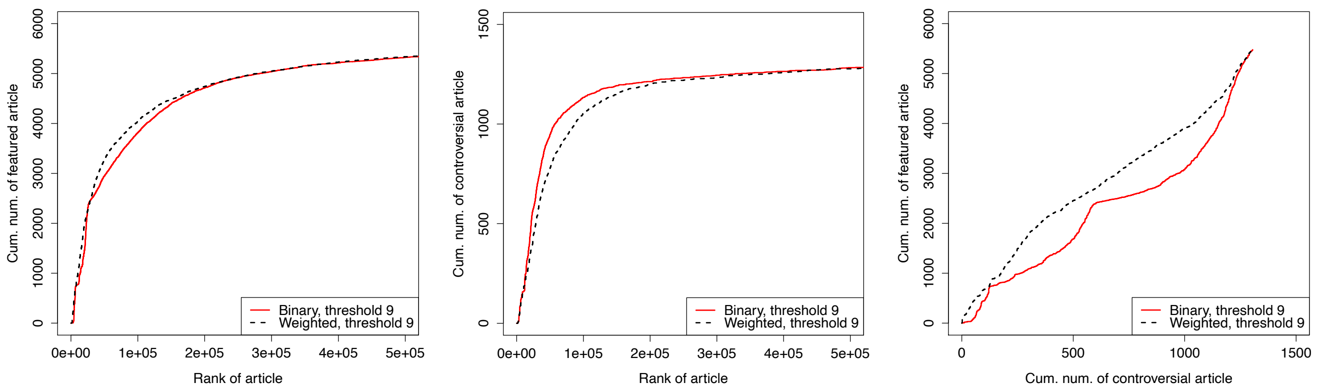

Figure 7. Performance of complexity measure for the binary network B and the weighted network W. (Left) Cumulative number of "featured" articles contained in the top-ranked articles in each complexity measure. (Middle) Cumulative number of "controversial" articles contained in the top-ranked articles in each complexity measure. (Right) The relation between the cumulative numbers of "featured" and "controversial" articles, which shows the performance of complexity measure to find "featured" articles without picking "controversial" articles.

Table 3. Top 20 articles in degree rank.

Degree Rank: r_{k_a}	Article title	Featured	Good	Controversial	Popular	(Eigenvector Centrality Rank)
1	Barack Obama	✓	–	✓	✓	1
2	Donald Trump	–	–	✓	✓	2
3	Wikipedia	–	–	✓	✓	28
4	United States	–	✓	✓	✓	12
5	George W. Bush	–	✓	✓	–	10
6	Adolf Hitler	–	✓	✓	✓	37
7	Michael Jackson	✓	–	✓	✓	20
8	Chicago	–	–	✓	–	6
9	London	–	✓	–	✓	19
10	Paris	–	✓	–	–	5
11	Dwight D. Eisenhower	–	–	–	–	17
12	Frank Sinatra	–	✓	–	–	4
13	David Bowie	✓	–	–	✓	11
14	Bill Clinton	–	✓	✓	–	118
15	World War II	–	✓	✓	✓	120
16	Pope John Paul II	–	–	✓	–	31
17	Los Angeles	–	–	–	–	38
18	Winston Churchill	–	–	–	–	81
19	New York City	–	–	–	✓	63
20	Vladimir Putin	–	–	✓	–	21

Table 4. Top 20 articles in Eigenvector centrality rank.

Eigenvector Centrality Rank	Article title	Featured	Good	Controversial	Popular	(Degree Rank)
1	Barack Obama	✓	–	✓	✓	1
2	Donald Trump	–	–	✓	✓	2
3	Portugal	–	–	–	–	29
4	Frank Sinatra	–	✓	–	–	12
5	Paris	–	✓	–	–	10
6	Chicago	–	–	✓	–	8
7	Iraq War	–	–	–	–	23
8	Haiti	–	–	–	–	27
9	Norway	–	–	–	–	28
10	George W. Bush	–	✓	✓	–	5
11	David Bowie	✓	–	–	✓	13
12	United States	–	✓	✓	✓	4
13	Austin, Texas	–	–	–	–	94
14	Bob Dylan	✓	–	–	–	26
15	Atlanta	–	✓	–	–	49
16	Johnny Cash	–	–	–	–	31
17	Dwight D. Eisenhower	–	–	–	–	11
18	Mexico City	–	–	–	–	65
19	London	–	✓	–	✓	9
20	Michael Jackson	✓	–	✓	✓	7

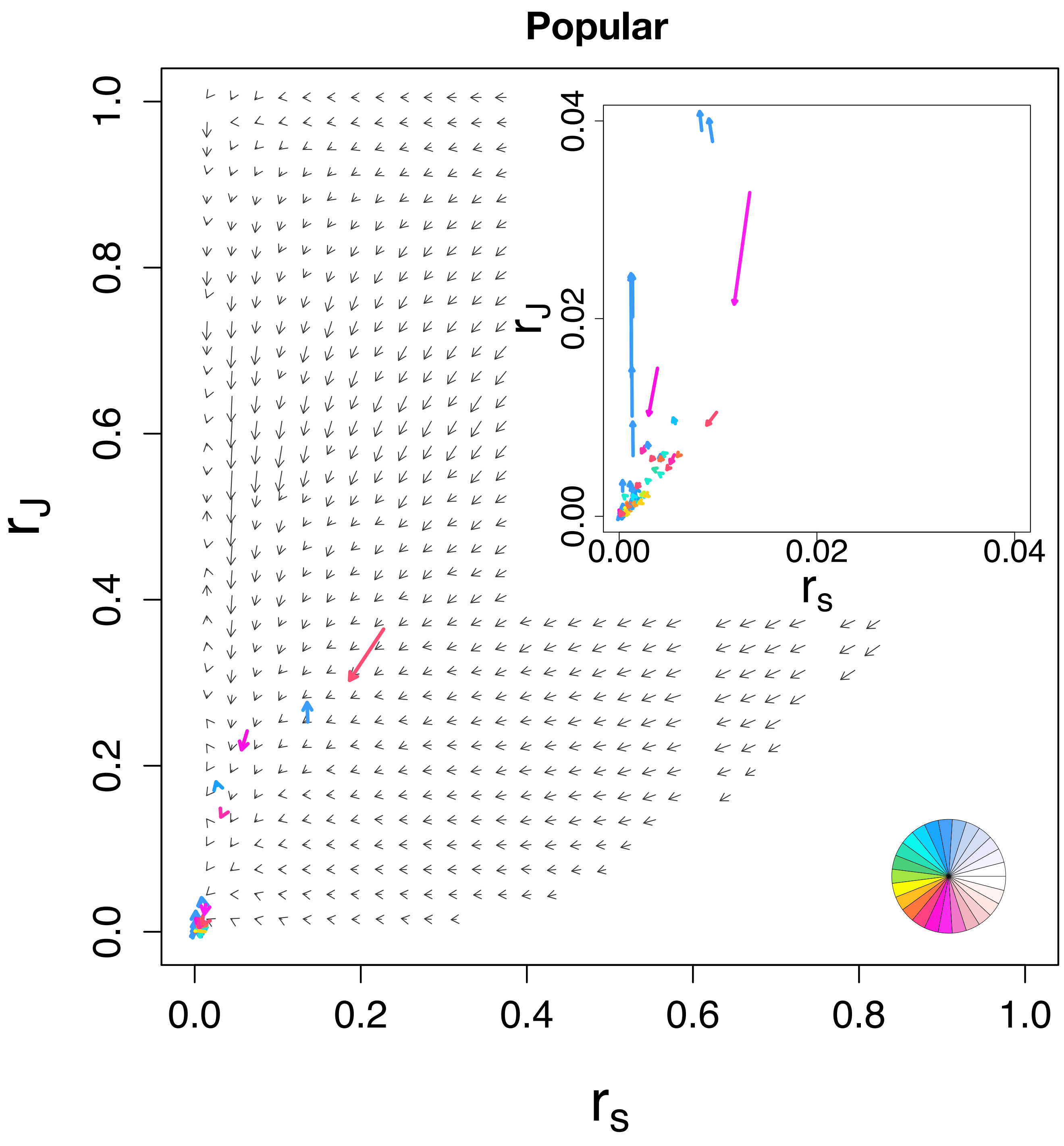

Figure 8. The temporal evolution of “popular” articles in the plane of relative ranks of the strength $r_s = R_s/N_a$, and of the complexity - strength rank ratio $r_J = R_J/N_a$. The flows of “popular” articles are overlaid on the average flow of all articles (grey).

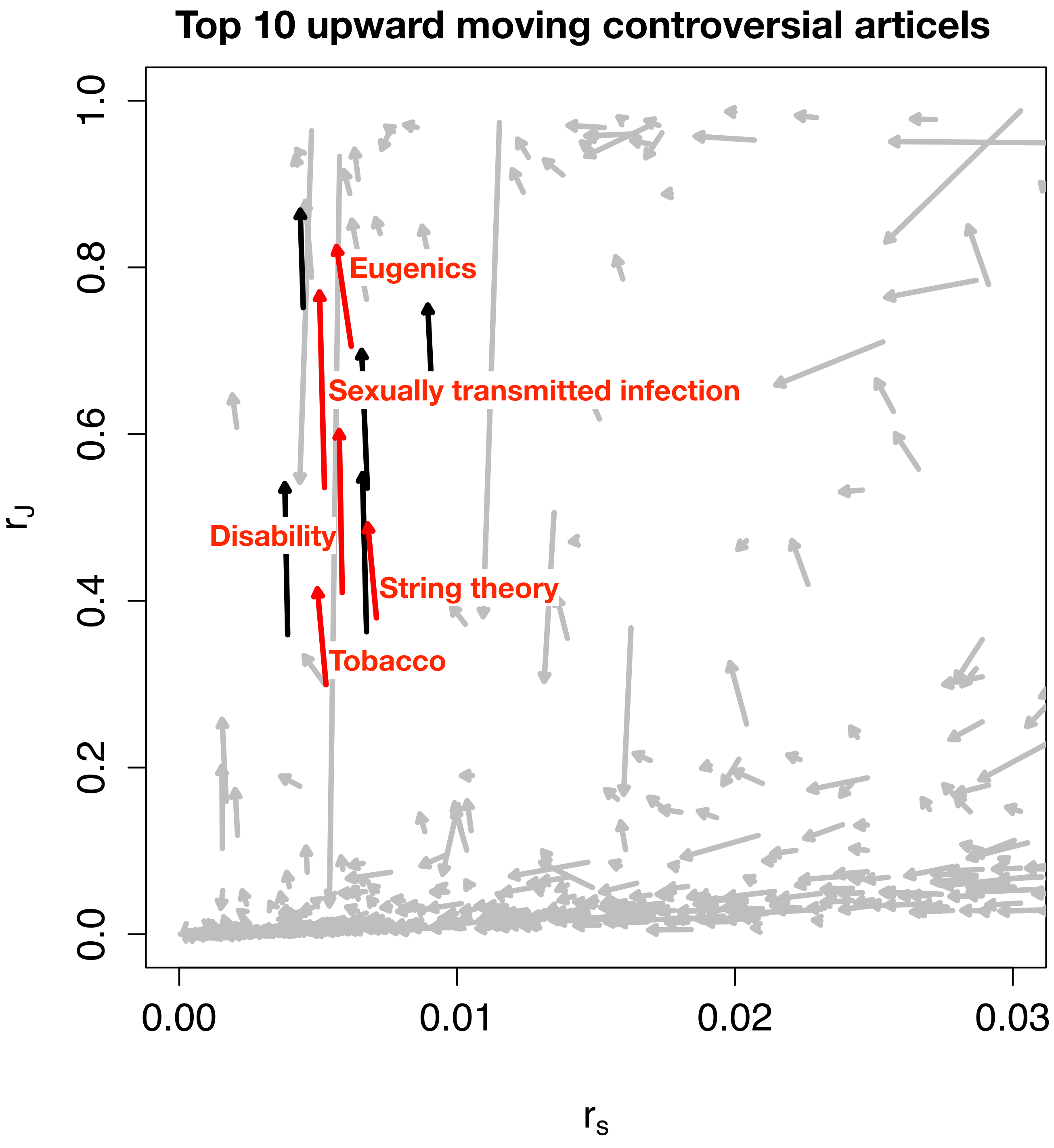

Figure 9. Top-10 upward moving “controversial” articles in the top 1% strength area in $r_S - r_J$ plane. Five out of these top-10 articles (red) are from “Science, Biology, and Health” category, while the share of this category in the whole “controversial” articles is about 10%. Top-10 articles are overlaid on the other “controversial” articles (gray).

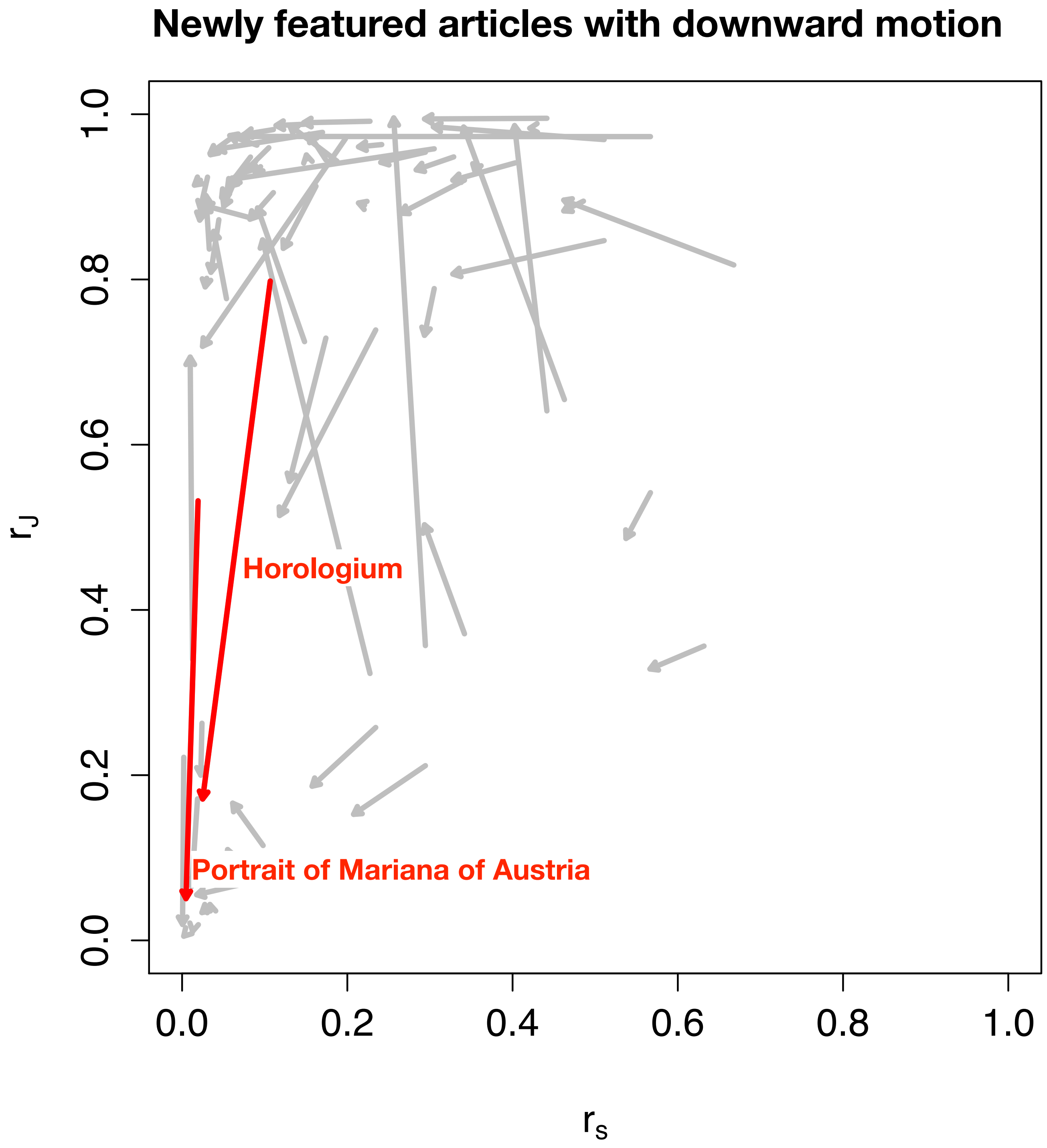

Figure 10. "Newly featured" articles with eye-catching downward motion (red) overlaid on the "Newly featured" articles (gray).

References

1. Jonhson, S., a, V. D.-G. & noz, M. A. M. Factors determining nestedness in complex networks. *PLoS ONE* **8**, e74025 (2013).
2. Tacchella, A., Cristelli, M., Caldarelli, G., Gabrielli, A. & Pietronero, L. A new metrics for countries' fitness and products' complexity. *Sci. Reports* **2**, 723 (2012).
3. Cristelli, M., Gabrielli, A., Tacchella, A., Caldarelli, G. & Pietronero, L. Measuring the intangibles: A metrics for the economic complexity of countries and products. *PLoS One* **8**, e70726 (2013).
4. Pugliese, E., Zaccaria, A. & Pietronero, L. On the convergence of the fitness-complexity algorithm. *Eur. Phys. J. Spec. Top.* **225**, 1893–1911 (2016).
